# Being parent of a child with congenital heart disease, what does it mean? A qualitative research

**DOI:** 10.1186/s40359-021-00539-0

**Published:** 2021-02-19

**Authors:** Nahid Dehghan Nayeri, Zahra Roddehghan, Farzad Mahmoodi, Parvin Mahmoodi

**Affiliations:** 1 School of Nursing and Midwifery, Tehran University of Medical Sciences, Tohid Square, Tohid Square, Nosrat St, Tehran, Iran; 2 Medicine Faculty, Kurdistan University of Medical Sciences, Kurdistan, Iran

**Keywords:** Life experiences, Parents, Heart disease, Pediatric

## Abstract

**Background:**

Childbirth is one of the invaluable human experiences and is associated with parental happiness. However, when a child is born with congenital heart disease, it creates emotional and mental distress. As a result, it changes the parents’ response to their child birth. Exploring parenthood experiences add to the body of knowledge and reveal new perspectives. In order to make healthcare professionals able to support these children and their families, they should first understand the meaning of this phenomenon. This study aimed to explore the meaning of parenting a child with Congenital Heart Disease in Iran.

**Methods:**

A qualitative study was adopted with a conventional content analysis approach and constant comparative analysis. Participants in this study were 17 parents, including parents of children with congenital heart disease who were selected by purposeful sampling method. Semi-structured interviews were used for data collection and continued to data saturation. Data were analyzed via MAXQDA 10 software.

**Results:**

Four categories and twenty three subcategories emerged as meaning of parenting a child with Congenital Heart Disease. Categories include “Emotional breakdown”, “The catastrophic burden of care”, “Spiritual beliefs of parents” and “The hard road”

**Conclusions:**

Fully understanding the life experience of these families will allow the implementation of targeted health interventions. Hence, by understanding the meaning of parenting a child with Congenital Heart Disease, healthcare professionals can asses parents emotional statues, information and spiritual needs, financial condition, insurance and marital status using CHD standards so that support is individualized, sensitive and time appropriate.

## Background

Congenital Heart Disease (CHD) is the most common type of birth defects [1]. Despite recent advances in the treatment of these patients, CHD remains the first cause of death in patients with congenital malformations [2]. The development of diagnostic technology has led to an increase in the number of newborns diagnosed with CHD [3].

There are different manifestations in children, depending on the type of CHD [1]. The manifestations observed in these children, the natural needs of the child and the conditions of the mother during the postpartum period create a stressful situation [4]. Numerous studies have found that parental role for the child with CHD is associated with a various socio-economic, physical, and psychological issues [3]. High level of distress is manifested in several ways including depression, anxiety, stress and somatization. Generally, parents of infants with CHD experience significantly higher parenting stress than parents of healthy peers on multiple subscales [5]. The parenting stress experienced by parents of infants with CHD is even higher than those of infants with other chronic healthcare needs [6].

Quantitative studies are useful when there is knowledge about the phenomenon in question and structured tools are used for their measurement. Hence, the qualitative research may help explore the meaning of parenting of a CHD child. However, every little study that has been done has only focused on a small part of the parent's and children’s life and has not been holistic [7].

A review of the literature shows that most studies on CHD have been conducted quantitatively. They have measured the distress, hopelessness and quality of life of parents. Some have examined the effects of living with a child with CHD on dimensions of mother and family that have not been addressed in qualitative studies [8]. For example, in a cohort study conducted by Solberg et al. (2012), they found that mothers of these children were at risk of mental illness [9].

Most of the qualitative studies have also been conducted either on mothers only with the aim of discovering the meaning of support from mothers' views or on patients rather than their parents [10, 11]. Sometimes they have studied a particular stage of the life process such as childhood, adolescence, or adulthood [4]. Qualitative studies have also been conducted in countries that differ in socioeconomic status, culture religion, health care system, diagnostic and treatment facilities, and people's belief in abortion with Iran [12]. Therefore, the lack of available knowledge on the parenting of these children in Iran has increased the need for this study.

Considering the significant number and increasing diagnosis of these children, understanding the meaning of this phenomenon is vital to identify the emotional, psychological, physical and economic needs of their families. On the other hand, how parents live with these children is a multidimensional, context based concept [13]. Therefore, this study aimed to explore the meaning of being parent of a child with CHD in Iran.

## Methods

### Study design & participants

This study is part of a PhD thesis. A qualitative study with content analysis approach and constant comparative analysis was applied to explore the meaning of being parent of a child with CHD. Parents who had a child with CHD were selected through purposeful sampling method as participants. Maximum variation in terms of parental age, education and child rank was used. Inclusion criteria included parents with children under 12 and having no primary congenital abnormalities except for CHD (Not having any congenital abnormalities of other systems except heart). Seventeen parents participated in the study. Demographic information of the participants is presented in Table 1. The guideline of consolidated criteria for reporting qualitative research (COREQ) was used for providing this manuscript.

**Table 1:** Demographic profile of the participants (n = 34)

Participant	Gender and Age	Child’s Age group
P1	Female in her 20 s	Neonate
P2	Male in his 20 s	Neonate
P3	Female in her 20 s	Infant
P4	Male in his 20 s	Infant
P5	Female in her 30 s	Toddler
P6	Male in his 40 s	Toddler
P7	Female in her 30 s	Toddler
P8	Male in his 30 s	Toddler
P9	Female in her 30 s	Infant
P10	Male in his 30 s	Infant
P11	Female in her 20 s	School age
P12	Male in his 20 s	School age
P13	Female in her 20 s	Neonate
P14	Male in his 30 s	Neonate
P15	Female in her 30 s	School age
P16	Male in his 30 s	School age
P17	Female in her 30 s	Pre-school
P18	Male in his 30 s	Pre-school
P19	Female in her 30 s	School age
P20	Male in his 30 s	School age
P21	Female in her20s	Pre-school
P22	Male in his 20 s	Pre-school
P23	Female in her 20 s	Toddler
P24	Male in his 20 s	Toddler
P25	Female in her 20 s	Pre-school
P26	Male in his 20 s	Pre-school
P27	Female in her 20 s	School age
P28	Male in his 20 s	School age
P29	Female in her 20 s	School age
P30	Male in his 20 s	School age
P31	Female in her 30 s	School age
P32	Male in his 40 s	School age
P33	Female in her 30 s	Pre-school
P34	Male in his 30 s	Pre-school

### Data collection

Data were collected using semi-structured interviews. The interview was developed for this study. The interviews usually began with a general question: "How did you feel when your child was diagnosed with this disease?” and other questions were: "What did your child’s heart disease mean for you?" Further explanations were also obtained by asking complementary probing questions such as ‘Would you please detail your explanation?’ and ‘explain your experience more, please’.

Mutual agreement of interviewees and researchers determined the time and place of the interview sessions. The interview time was 30 to 95 min and on average 45 min for each interview. A digital device was used for recording the interviews. The interviews were continued until data saturation, where categories and subcategories were completed and no new category was obtained [14].

### Data analysis

Content analysis approach and constant comparative analysis was used for data analysis. Each interview was considered as a unit of analysis. The interviews were recorded and transcribed verbatim and read repeatedly. Then, the data were broken down into units of meaning that were extracted from the statements and labeled with conceptual names. Then codes were compared based on similarities and differences and grouped into categories. Each subcategory with similar mean was grouped as categories and categories were grouped as main categories [15]. MAXQDA 10 was used to manage the textual data.

### Trustworthiness

This study applied the criteria suggested by Guba and Lincoln to evaluate the credibility of the data [16]. The prolonged engagement with the participants during the interview period helped to establish trust, better understanding, and a close relationship with the participants. Moreover, analytic categories, interpretations, and conclusions were tested using member checks. Detailed descriptions of contexts and participants were used for transferability.

## Results

Standards for Reporting Qualitative Research (SRQR) were used for reporting the results. The meaning of parenting a child with a CHD consisted of four main categories: "Emotional breakdown", "The catastrophic burden of care", "Turn to spirituality" and "The hard road” (Table 2).

**Table 2:** Meaning of being parent of a child with CHD

Themes	Major Subcategory	Subcategory
Emotional breakdown (n = 28)	Denial (n = 25)	
	Shock (n = 23)	
	Sadness (n = 21)	
	Isolation (n = 20)	
	Feeling guilty (n = 17)	
	The most difficult moment in parenting life (n = 15)	
The catastrophic burden of care (n = 25)	Suffering from child care (n = 24)	Constant self-sacrifice (n = 23)
		Restriction (n = 22)
		Painful waiting (n = 20)
		Exhausting search (n = 18)
		Suffering from the judgment and curiosity of others (n = 15)
		Delayed vaccination and circumcision stress (n = 15)
	Being responsible for diagnosis, treatment and child care (n = 20)	Parental distress over the child being hospitalized (n = 19)
		Anxiety related to invasive procedure (n = 18)
		Mental turbulence (n = 17)
		Taking care of other disease of the child (n = 17)
	The need to strive to provide the best medical care (n = 19)	
Turn to spirituality (n = 21)	Feeling the orphans and helping the poor (n = 20)	
	Trust in God (n = 17)	
	To undergo a divine test (n = 16)	
The hard road (n = 19)	Emotional separation of parents (n = 18)	
	Harsh living conditions (n = 17)	

### Emotional breakdown

Emotional breakdown is indeed the reaction and feelings of the parents involved in this study at the time of diagnosis which includes "denial", "sadness", "isolation", "shock", "feeling guilty " and "the most difficult moment in parenting life."

#### Denial

For some parents, a child's CHD can be a crisis. Therefore, they deny it and do not seek treatment or seek confirmation of diagnosis by several doctors. "My husband said our baby is healthy and don’t need to seek treatment. The doctors are wrong" said a mother (P12).

#### Shock

Getting shocked can be understood from sentences like "it sounds like somebody poured a bucket of water over my head", "when I find out my baby has a heart disease my legs broke" "I died and lived again", "I didn't know how my day and night are passing" and "I couldn't walk on my feet". "The first time I was told about my child’s disease I was unconscious for an hour and a half” said a mother (P9**).**

#### Sadness

Parental grief can be understood from the constant cries, impatience and dying parental life. Sometimes due to a history of abortion, this concern is heightened. “When I heard the news of my child’s illness, my face was burning of heat and I had to get out of the doctor’s office to take a breath” (p3).

#### Isolation

Some parents avoid communicating with others and isolate themselves from the community after being informed of a child's CHD. "I didn't talk to anyone for a long time and I was just by myself. I didn't want to see anyone", said a mother (P5).

#### Feeling guilty

Parents often feel guilty by not diagnosing child's CHD during pregnancy, not seeking of modern health care facilities earlier and giving birth to the child. "We were upset that we didn't understand my son's illness," said a father (P12).

#### The most difficult moment in parenting life

Regardless of how parents interpret their child’s heart illness, the way they react to, it is the most difficult moment in their parenting life. "The doctor said his heart had a hole. As I stood there, my legs were loose. I don’t think that in all my life I could ever experience the gravity of my son's illness” said a father (P14).

### The catastrophic burden of care

Caring for a child with CHD is difficult due to specific nutritional, environmental, sleep and medication care. This category comprises the three sub-categories of "suffering from child care", "being responsible for diagnosis, treatment and child care" and "the need to strive to provide the best medical care".

#### Suffering from child care

This category includes six subcategories of constant self-sacrifice, restriction, painful waiting, exhausting search, suffering from judgment and curiosity of others, delayed vaccination and circumcision stress.

##### Constant self-sacrifice

Self-sacrifice means having others take precedence over oneself. Based on the experiences of the participants, parents sell their vital organs to cover the cost of their child's treatment, or take them to modern health centers despite the severe financial poverty. "Sometimes when we would go to the doctor, we would buy food for my son, but my husband and I would go back home hungry” said a mother (p17).

##### Restriction

Caring for a child with CHD includes avoiding exercising, chest trauma, exciting games, crying, being stressed out, weight-lifting, entering crowded environments, and post-surgical care. All of the above limit the parents. "In the past six years that my baby has been sick, we have not gone on a walk or a wedding," said a mother (P27).

##### Painful waiting

In some cases, the baby's weight must reach a certain level to allow for heart surgery. On the other hand, in these children, weight gain is usually very slow. So this period of time for parents is associated with expectation and anxiety. "The doctor said we had to wait until he weighs about 9 kg then we would conduct surgery but he couldn't eat anything," said a mother (P11).

##### Exhausting search

Lack of awareness and ambiguities forces parents to inquire about the symptoms of children with CHD and then compare their child's symptoms. One mother said: "I'm going to the house of those who have this problem. I ask what you did. For example, I ask the surgeon's name. One had the same problem like my daughter and got married. I even went to her house"(P11).

##### Suffering from the judgment and curiosity of others

Diagnosing a child with illness by others based on his or her appearance is a discomfort to the parent, as people look for the cause as soon as they notice it. Finally, others sometimes judge parents with curiosity and accuse them of incapability to have a healthy child. " One of our guests asked that your baby is a boy so why have you polished her nail. We have not done so but his nails were so bruised shed though we have done so” said a father (P16).

##### Delayed vaccination and circumcision stress

One of the concerns of parents for months is delaying their child's vaccination and circumcision due to CHD. "We took his 2^nd^ month vaccination in the 4^th^ month" said a mother (P7).

#### Being responsible for diagnosis, treatment and child care

Consecutive hospitalizations, frequent visits, aggressive procedures, nutritional, environmental and medicinal precautions, and caring for other illnesses (if the child has another illness concurrently) make it difficult to care for these children.

##### Parental distress over the child being hospitalized

Parents are often suffer from anger and guilt because of unawareness of the cause and process of treatment and care, consecutive visits, economic consequences of child’s illness, child suffering during illness, separation from the child, lack of knowledge about the future of the disease, disordering life and routine family activities. "When he needed surgery, we were in hospital for over one month with my child alone. I hoped to have one hour to take a rest somewhere. My feet were inflamed and I wished to take the shoes off” said a mother (P13).

##### Anxiety related to invasive procedure

All children respond to pain get excited, irritable, and restless, even have nightmares, sleep and feeding disturbances. On the other hand, parents suffer greatly from pain, anesthesia, its complications, and as the result of aggressive procedure. One father said: "My kid has angiography for the second time. It took me three hours while I was waiting for the angiography. I was dead and alive 300 times in that 3 h (P18).

##### Mental turbulence

Children with CHD often face the slow process of weight gain. Therefore, parents pay special attention to the number of times they are fed and the type of their diet. On the level of activity, they may also deprive the child of emotions, heavy activity and running. Daily use of tablets and syrups is also a routine for most children. "We wouldn't let him cough or sneeze or have a hit on his back because he could break the wall between his atriums again" a mother said. (P9).

##### Taking care of other diseases of the child

Having another disease simultaneously with CHD increases parents’ suffering. "When she was born, she had choanal atresia, in addition to CHD. So we had to suction her nose permanently" said a father. (P18).

#### The need to strive to provide the best medical care

Parental experiences show that nothing can prevent the provision of the best medical care for the child, even if the parents are poor and illiterate. Often, mothers sell their jewelry or their child’s jewelry or home furnishings, borrow from others to take their child to the capital's health centers to make sure they do everything they can to improve their child. “My husband was going to sell home furnishings to make money to take our son to Tehran to see a good surgeon," said a mother who had a worker husband. (P19).

### Turn to spirituality

Parents have a different perception of their child's CHD, depending on their cultural background. Some people call a child's CHD a divine test. They hope to survive and improve their child by feeding the orphans, helping the poor and trusting in God.

#### Feeding the orphans and helping the poor

A number of parents start doing humanitarian work in the hope that God will preserve and heal their child. The responsibility of caring for an orphaned child, providing food for the poor, sacrificing for the needy are among the things parents do to protect their child. "I also took care of a kid whose father was dead so that God help my child." Said a father.(P12).

#### Trust in God

Trust means letting things to be managed by God. All participants in this study were Muslim. Many of them believed that God will decide for their child's death or life. "If God wants this child will survive” said one father. (P18).

#### To undergo a divine test

Some parents believed their child's illness to be a test of God. A mother said, "That kid is not sinful that God created him with CHD. It's just a divine test for me and his father. Do we have patience or not?" (P17).

### The hard road

Participants believe that the birth of a child with CHD is associated with anxiety, suffering, fear, economic, emotional, and marital problems. Whenever this occurs under conditions such as emotional separation from parents and harsh living conditions, the severity of this psychological distress will increase. In fact, the hard road means the context is the underlying parental life at the time a child with a CHD was born.

#### Emotional separation of parents

Some of the participants in this study were not satisfied with the quality of the marital and emotional relationships due to the infidelity of their husbands or high age differences and thus had emotional separation. They had a child with CHD in such a condition. "We are living together, but we are separated," said a mother. (P33).

#### Harsh living conditions

Mother doing heavy farming and agricultural activities, earthquake coinciding with cesarean section of the mother, child birth coinciding with her father being indebted, living in a humid house and near animal waste disposal (causing the child to develop asthma), all indicate the difficult living conditions of some of the participants. "My son grew up in a dinghy humid house." a mother said. (P11).

## Discussion

This study explored the meaning of parenting a child with CHD. Emotional breakdown, the catastrophic burden of care, turning to spirituality and the hard road are the meaning of parenting a child with CHD from the parents’ perspective who participated in this study. The category of emotional breakdown includes subcategories of denial, shock, sadness, isolation, feeling guilty and the most difficult moment of parenting.

One of the most important things that parents experience when living with a CHD child is parents’ feeling at the time of diagnosis which is a critical moment [17]. The parents in this study said they were shocked and saddened at the time of diagnosis of their child. They were then unable to accept their child's illness, so they sought to deny it with their isolation, which was not mentioned in other studies. On the other hand, they blamed themselves for their child's illness and suffered from feeling guilty. All parents mentioned the most difficult moment in their lives was the diagnosis moment.

A similar study reported that shock was described by all parents receiving a postnatal diagnosis but half of those receiving an antenatal diagnosis [18]. Other initial feelings described by parents included bewilderment, their general emotional state and concern for their child and a kind of transition from being shocked at first to being blessed at end [6, 17]. Parents may experience guilt about the condition, leading to reduced self-esteem and parental competence.

Fear and stress can lead to the developmental of acute stress disorder (ASD) and post- traumatic disorder (PTSD), whereas social support decreases stress [19]. The children of parents suffering from PTSD have a greater risk of developing sleep and eating disorders with increased consequences in the number of hospital access [20].

The CHD standards and service specification document (NHS England 2016:149) has outlined national standards for ongoing care at the time of diagnosis. This includes a specific standard that states a psychologist must be available to support families and children at any stage in their care but particularly at the stage of diagnosis [18].

Difficulties of caring process were another experience of parents which includes subcategories of constant self-sacrifice, restriction, painful waiting, exhausting search, suffering from the judgment and curiosity of others and delayed vaccination and circumcision stress.

Parents are faced to bitter realities in taking care of their child unexpectedly. Howe ever, in many cases they were not able to do anything about them. What was extracted from the experiences of the participants in this study is that the routine parenting life is affected by the difficult nature of the child's care, including frequent hospitalizations and conditions associated with it. Therefore, parents are restricted from doing many social activities. This finding is consistent with the results of some studies which showed routine visits, alteration in medications and limitation in activities were effective in making mental disorders in both patients and their families [21].

The parents of this study consistently sought out research on CHD and people with it. They suffered from witnessing their child symptoms and are self-sacrificing to look after them. However, the parents of this study despite financial poverty, tried to get their child to the best medical centers for the best medical care. These are not mentioned in qualitative studies in other fields.

Several studies of parents of children with CHD have found that many seek information even using the Internet and sometimes relying on inaccurate sources. These findings highlight the importance of communicating with these parents and accurately assessing their knowledge level to provide them with accurate, timely, and useful information [22].

CHD has resulted in physical limitations for these children, which subsequently decrease the quality of family life and affect the child's social and emotional freedom [16]. The results of a study showed that parents of children of over 2 years and older have difficulty managing child behavior in terms of the physician-defined limitations and attracting child’s cooperation [23]. Their children also hate social exclusion because of their sense of belonging to their peers [24].

Another consequence of CHD child is the turning to spirituality. Parents may consider a child's heart disease to be a divine test and rely on God to improve the child. In this respect they engaged in humanitarian work that has not been seen in other studies.

In another study the improvement of the spiritual life of the mother’s were reported. They named referring to the religion as taking refuge from anxiety. These show the imaginable advantages of spiritual support in managing families with patients suffering from CHD [23]. Contrary to these findings, in another study, the mothers witnessed how their child prayed that God would heal them so that they would play like their peers and no one would worry about them. They witnessed how their child wished to free from the nightmare of needles and hospitalization. These factors decreased the spirit of the mother [21].

The hard road is the last finding of this study. Mother doing heavy farming and agricultural activities, earthquake coinciding with cesarean section of the mother, child birth coinciding with her father being indebted, living in a humid house and near animal waste disposal, financial poverty and lack of parental awareness were cited by the study participants as a difficult background of life at the time of diagnosis of a child with CHD which has not been mentioned in other studies.

## Conclusions

Taking good care of the parents is an investment in the shared future of the child and the family. Through the results of this study, the normal reactions of parent, their emotional and spiritual needs, the catastrophic burden of care and the hard road of the families’ life were revealed. Fully understanding the life experience of these families will allow the implementation of targeted health interventions. Hence, healthcare professionals need to asses parents emotional statues, information and spiritual needs, financial, insurance and marital status through CHD standards so that support is individualized, sensitive and time appropriate. Since, qualitative researches are fundamental, the knowledge obtained from this study can reveal the meaning of parenting for children with CHD. This knowledge can give nurses insight to be used in family-centered cares.

## Data Availability

The datasets used and analyzed during the current study are available from the corresponding author on reasonable request.

## Abbreviations

CHD: Congenital heart disease
PFO: Patent foramen ovale
ASD: Atrial septal defect
VSD: Ventricular septal defect
PDA: Patent ductus arteriosus
PTSD: Post-traumatic stress disorder
COREQ: Consolidated criteria for reporting qualitative research
SRQR: Standards for reporting qualitative research
TUMS: Tehran university of medical sciences
P: Participant
